# Ragweed (*Ambrosia artemisiifolia*) pollen allergenicity: SuperSAGE transcriptomic analysis upon elevated CO_2_ and drought stress

**DOI:** 10.1186/1471-2229-14-176

**Published:** 2014-06-27

**Authors:** Amr El Kelish, Feng Zhao, Werner Heller, Jörg Durner, J Barbro Winkler, Heidrun Behrendt, Claudia Traidl-Hoffmann, Ralf Horres, Matthias Pfeifer, Ulrike Frank, Dieter Ernst

**Affiliations:** 1 Institute of Biochemical Plant Pathology, Helmholtz Zentrum München, German Research Center for Environmental Health, 85764 Neuherberg, Germany; 2 Botany Department, Faculty of Science, Suez Canal University, Ismailia, Egypt; 3 Biochemical Plant Pathology, Technische Universität München, Center of Life and Food Sciences Weihenstephan, 85350 Freising-Weihenstephan, Germany; 4 Research Unit for Environmental Simulation, Helmholtz Zentrum München, German Research Center for Environmental Health, 85764 Neuherberg, Germany; 5 Center of Allergy & Environment München (ZAUM), Technische Universität and Helmholtz Zentrum München, 85764 Neuherberg, Germany; 6 CK-CARE, Christine Kühne – Center for Allergy Research and Education, Davos, Switzerland; 7 GenXPro GmbH, 60438 Frankfurt am Main, Germany; 8 Institute of Bioinformatics and Systems Biology, Helmholtz Zentrum München, German Research Center for Environmental Health, 85764 Neuherberg, Germany; 9 Institute of Environmental Medicine, UNIKA-T, Technische Universität München, Munich, Germany

**Keywords:** *Ambrosia artemisiifolia*, Allergen, Allergy, CO_2_, Drought, Flavonoids, Pollen, Ragweed, Scanning electron microscopy, Transcriptome

## Abstract

**Background:**

Pollen of common ragweed (*Ambrosia artemisiifolia*) is a main cause of allergic diseases in Northern America. The weed has recently become spreading as a neophyte in Europe, while climate change may also affect the growth of the plant and additionally may also influence pollen allergenicity. To gain better insight in the molecular mechanisms in the development of ragweed pollen and its allergenic proteins under global change scenarios, we generated SuperSAGE libraries to identify differentially expressed transcripts.

**Results:**

Ragweed plants were grown in a greenhouse under 380 ppm CO_2_ and under elevated level of CO_2_ (700 ppm). In addition, drought experiments under both CO_2_ concentrations were performed. The pollen viability was not altered under elevated CO_2_, whereas drought stress decreased its viability. Increased levels of individual flavonoid metabolites were found under elevated CO_2_ and/or drought. Total RNA was isolated from ragweed pollen, exposed to the four mentioned scenarios and four SuperSAGE libraries were constructed. The library dataset included 236,942 unique sequences, showing overlapping as well as clear differently expressed sequence tags (ESTs). The analysis targeted ESTs known in *Ambrosia*, as well as in pollen of other plants. Among the identified ESTs, those encoding allergenic ragweed proteins (Amb a) increased under elevated CO_2_ and drought stress. In addition, ESTs encoding allergenic proteins in other plants were also identified.

**Conclusions:**

The analysis of changes in the transcriptome of ragweed pollen upon CO_2_ and drought stress using SuperSAGE indicates that under global change scenarios the pollen transcriptome was altered, and impacts the allergenic potential of ragweed pollen.

## Background

Pollen of the common ragweed (*Ambrosia artemisiifolia*) is a main cause of allergic diseases in Northern America [1,2]. This species is the most widespread *Ambrosia* and the weed has become spreading as a neophyte in Europe, and will become a serious health problem in sensitized populations [3]. The distribution of ragweed in Europe began approximately 100 years ago and is currently primarily found in the Rhône valley, Hungary, Croatia, Bulgaria, Northern Italy and Eastern Austria, but it is also spreading in Germany [4,5] (http://www.ambrosiainfo.de/ 53223897640d5c602/ index.html).

So far, the allergenic proteins of ragweed can be arranged into six biological groups [3,6]. Approximately 48 allergenic proteins are known for the genus *Ambrosia*, and 32 proteins, including multiple isoforms, are known for *A. artemisiifolia* (http://www.allergome.org). The major allergen of ragweed is Amb a 1, an acidic non-glycosylated 38-kDa protein consisting of a 26-kDa α-chain and an associated 12-kDa β-chain [3].

It is hypothesized that climate change and air pollution will affect the allergenic potential of pollen, either by a changed pollen season, by a changed pollen amount, by changes of the surface exine or by directly increasing the allergenic transcripts and proteins and interactions with biologically important ligands, e.g., flavonoids [2,7-11]. Studies on effects on climate change on respiratory allergy are still lacking and only a few epidemiological reports on urbanization and air-pollution on pollen allergenicity are available [12]. An overview for risk factors on allergic disease discussing genetics aspects, indoor and outdoor pollution, socio-economic factors, climate change and migration has recently been published [12]. The proteomic profiling of birch pollen isolated from different sites indicated differences between allergenic and non-allergenic proteins [13]. In contrast, birch pollen isolated from an urban and rural site showed no difference in allergenic protein expression, indicating that allergenicity is determined by additional allergen carriers [14]. An in vivo study on birch pollen also sampled from different sites could correlate elevated ozone levels to higher allergenicity as well as to an increased allergen content [15]. It was recently shown that twice ambient ozone levels resulted in an increased content of allergenic proteins in two rye cultivars [16]. In ragweed, elevated ozone fumigation resulted in a changed transcriptional profile, including transcripts for allergenic proteins [17]. Elevated CO_2_ concentrations showed an increase growth of ragweed and its pollen production [18-21], and an increased content of Amb a 1 allergen was observed [22].

In addition to increasing CO_2_ concentrations, future atmospheric warming, as well as hot and dry summer periods are also expected [23,24]; IPPC Report 2007. Regulatory networks in cellular responses to drought, including abscisic acid-dependent and -independent systems, are well known during plant growth and development [25-30]. Regarding transcriptomic and proteomic analyses of pollen, literature reports have focused on different developmental stages of pollen, mature pollen and pollen germination [31-36]. Regarding temperature effects, differentially cold-regulated genes were detected in mature pollen of *Arabidopsis thaliana*[37].

Flavonoids are ubiquitous plant secondary metabolites and are important in plant development and reproduction, as well as in protection against abiotic and biotic stress factors [38,39]. The yellow color of pollen can be traced back to flavonoids, thus shielding the pollen genome from UV-B radiation [40]. In addition, flavonoids play a role in male fertility, and quercetin is an important germination-inducing compound in maize and petunia but not in *Arabidopsis* or parsley [41,42]. Flavonoids may be involved in the modulation of immune responses and thus may also be important in the allergenic response of pollen [43,44]. In human health, IgE-binding of allergens may be influenced by attached flavonoids [45,46]. The pathogenesis-related proteins (PRs) consist of a large group of homologous proteins in different plant species and many PRs are expressed in pollen and can act as allergens [47]. A direct interaction of birch PR-10c with biologically important molecules, including flavonoids, was shown by Koistinen et al. [48]. Similarly, flavonoids bind to the major birch allergen Bet v 1 [9], which also belongs to the PR-10 family [49]. Recently it was shown, that a quercetin derivative directly binds to the C-terminal helix of Bet v 1, and that this binding plays an important role during the inflammation response [50]. These results indicate that, in addition to allergenic proteins, additional allergenic carriers may also be involved in pollen allergenicity, which is not exclusively triggered by known allergenic proteins [14,51,52].

These studies suggest that global change will affect the allergenic potential of pollen and play a role in human health diseases related to allergic rhinitis and asthma. From this perspective, a transcriptome-wide analysis of the highly allergic pollen of ragweed would not only help in understanding climate impact on expressed pollen transcripts but also gain a deeper insight into the expected changes of pollen allergens. Flavonoids analysis will allow a better understanding of their possible function as additional allergenic carriers and also contribute to the relevant UV-B-absorbing metabolites of pollen. In a previous study, we showed that twice the ambient level of ozone resulted in a changed transcriptional profile of ragweed pollen, including encoded allergenic proteins [17]. In this study, we modified the global climate change approach by linking the transcriptional network changes of ragweed pollen to elevated CO_2_ concentrations and an extreme drought event. We highlight that the global change scenarios will affect the transcriptome of pollen and will also increase the abundance of allergen-related transcripts relevant for human health.

## Results and discussion

### Morphological parameters and pollen viability

Two main different leaf morphologies between the plants were observed: plants with strong pinnate leaves (i) and plants with only weak pinnate leaves (ii), as has been reported for ragweed with the same genetic background in exposure chambers [21].

Pollen viability was slightly reduced under elevated CO_2_ levels; however, this result was not statistically significant (Additional file 1). Similarly, it was shown that the pollen performance decreased in *Raphanus sativus* in response to elevated CO_2_ levels [53]. Drought stress resulted in a reduction of the pollen viability from approximately 46% to 24% (Additional file 1). The decreased pollen viability under drought stress is in accordance with several literature reports also demonstrating a reduced viability and pollen grain production [54-57]. Interestingly, this drought effect could be partially mitigated by elevated CO_2_ with a slight increase from 24% to 30% (Additional file 1), indicating no additive effects of elevated CO_2_ and drought.

### Secondary metabolites

**Figure 1:**
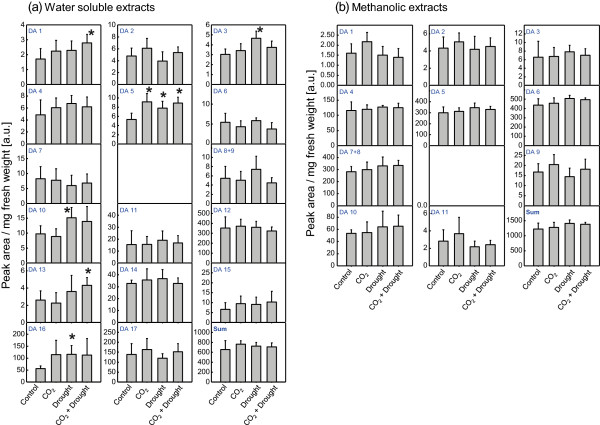
**Amount of PBS-soluble (a) and methanolic-extractable (b) phenolic metabolites in ragweed pollen.** The separation was performed by RP-HPLC. The bars (N = 5) indicate SD and significant differences are indicated by an asterisk.

Typical reverse-phase high-performance liquid chromatography (RP-HPLC) diagrams for water soluble metabolite extracts revealed 17 compounds, with the highest amounts in particular for metabolite 12 and 17, both are quercetin derivatives and methanolic extracts showed 12 different metabolites, congruent to data given by Kanter et al. [17] (Additional file 2). The total amounts of individual compounds for the final harvest are given in Figure 1. No significant changes could be observed between the control, elevated CO_2_, drought and elevated CO_2_ plus drought samples, similar to what has been described for ozone-treated pollen. However, individual metabolites of the PBS extract showed increased levels upon drought stress at both CO_2_ concentrations (Figure 1a; DA1, DA3, DA5, DA10, DA13 (quercetin derivative) and DA16 (kaempferol derivative). This change in individual metabolites is in contrast to pollen of ozone-fumigated ragweed that showed no change of such individual metabolites. Flavonoids have been shown to accumulate under drought stress in several plants, thus playing a physiological role in water tolerance and protection against oxidative stress [58-60]. Moreover, detailed analyses showed that the level of quercetin derivatives also increased upon drought stress in different plants [60-62], clearly indicating that in pollen of drought-stressed plants, the accumulation of individual flavonoid metabolites may play a protective role against oxidative stress and damage of the pollen tissue. Elevated CO_2_ resulted in increased levels of flavonoid metabolites in several plant species [63-65]. In ragweed pollen, the metabolite level was approximately at the same levels under drought, irrespectively of the CO_2_ concentration (Figure 1). Thus, drought might be more important than elevated CO_2_ in increasing the levels of these individual metabolites. A single metabolite (DA 5) was also increased under CO_2_ treatment alone (Figure 1), similar to the impact of CO_2_ in soybean, where the concentration of only one flavonoid, a quercetin glycoside, was also increased [66]. This result indicates species-specific CO_2_ responses in flavonoid content and composition [67,68].

### SuperSAGE dataset

**Figure 2:**
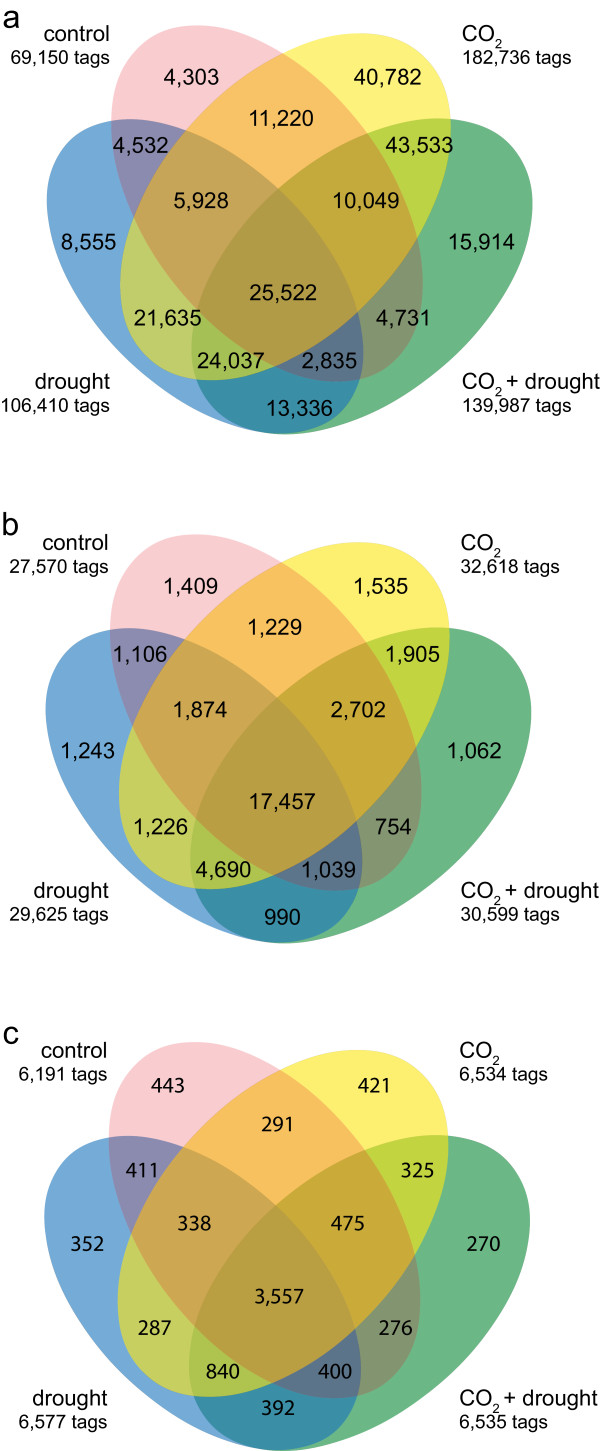
**Venn diagram.** Number of common and unique SuperSAGE sequence tags. For each sequence, the tag amount in the individual samples was analyzed. Sequences with ≥ 1 appearances in two, three or all of the samples are shown by individual overlapping regions. The total number of sequence tags per library is indicated. **a** reflects the distribution of sequence tags in the original dataset. **b** gives the distribution of sequenced tags filtered for tpm > 0.8. **c** indicates the sequence tag distribution for a stringently filtered dataset with the following criteria: tpm > 0.8; score of BLAST hit > 40; and removal of sequence tags without BLAST result (“no hit”).

**Figure 3:**
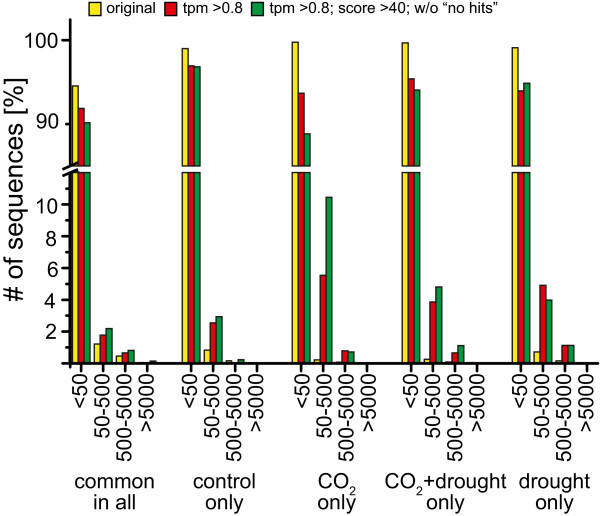
**Distribution of low- to very high-abundant sequence tags.** The tags were found uniquely under control conditions (380 ppm CO_2_), under elevated CO_2_ (700 ppm CO_2_), under CO_2_ plus drought or under drought, or found to be common in all four libraries at one time. The data were analyzed for the different filter criteria indicated in the graph.

The number of sequenced tags ranged from approximately 4.5 × 10^6^ to 17.2 × 10^6^ in the four libraries (Additional file 3, Info). The tag frequencies are given in Additional file 3 (All_Libs20101207). The SuperSAGE dataset included 236,942 different non redundant sequences (tags) of 26 bp in length (Additional file 3, All_Libs20101207). For each of these sequences (tag), the tag amounts are provided and count how often a unique sequence was found in each of the four libraries. One sequence (tag) can be found in one, two, three or all four of the libraries, as indicated in the overlapping regions in Figure 2a but, according to the transcript expression, in different quantities (tag amounts). The sequenced tag counts for each unique sequence in all of the libraries ranged from ≤ 50 (low), 50–500 (mid), 500–5000 (high) and ≥ 5000 (very high) (Table 1). The normalized values of each tag in relation to 10^6^ tags (tpm) for each library resulted in approximately 99.5% of low- and mid-abundant unique tags, while high- and very high-abundant tags represented only approximately 0.2% - 0.4% (Table 1). A similar distribution of abundant classes has also been reported for other SuperSAGE libraries [69-71]. The four libraries had approximately the same unique sequences for the very high-abundant class (31–37), the high abundant class (239–270) and the mid-abundant class (863–1129). In contrast, the low-abundant class was more variable, reflecting also the total number of unique sequences of each library (Table 1). According to the cumulative frequency distribution, only those tpm values greater than 0.6 to 0.8 can be considered expressed [72] (Additional file 4). Therefore, transcripts with a tpm threshold < 0.8 were eliminated, resulting in more stringent values, coming up with 40,221 unique sequences (Figure 2b). Finally, we eliminated all of the sequences with the description ‘no hits’ and the score of the BLAST hit was set to ≥ 40. These parameters resulted then in 9,078 unique sequences and an equal distribution in all 4 of the libraries (Figure 2c). The low-abundance sequences were strongly reduced in all of the libraries to approximately 90.0%, whereas those sequences in the mid- and high-abundant groups strongly increased up to 10% (Table 2, Figure 3). Additionally, MapMan was used to group the SuperSAGE tags into several functional categories (BIN-codes) [73]. For this grouping, the SuperSAGE tags were matched to *Ambrosia* 454-transcriptome data (contigs + singletons) [17]. The data were then BLASTed against Arabidopsis (TAIR) to identify *Arabidopsis* homologues, which then could be sorted to the BIN-codes (workflow: Additional file 5) and only log_2_-fold changes of at least 1.5 were further examined (Additional file 6). Interestingly, elevated CO_2_ + drought conditions resulted in higher log_2_-fold changes compared to the single treatments, indicating additive effects. Transcripts with homologies to abiotic stress were mainly up-regulated under all three scenarios, including also dehydration-responsive transcripts, heat-shock proteins and chaperones. Regarding drought stress, this result is not surprising and has also been reported in the literature [26,30,74,75]. For the BIN-name cell wall, a pectate lyase family member and expansin were clearly up-regulated. Pectate lyases are important for pollen tube growth by pectin degradation. However, in ragweed pollen, pectate lyases belong to the major allergen Amb a 1 family (AllFam database; http://www.meduniwien.ac.at/allergens/allfam/chart.php?kingdom=Plants&exposure=Inhalation&list=10&page=0). Expansins are important for the pollen tube and for cell wall changes and confer drought tolerance [76,77]. Moreover, expansins also belong to pollen allergens (AllFam database). The most strongly up-regulated transcript (*CER1*) in all three of the treatments is involved in wax biosynthesis (log_2_-fold 5.3 - 9.2). *CER1* is mainly expressed in inflorescences and siliques and is induced by osmotic stress [78]. This result demonstrates that wax biosynthesis is enhanced under climate change scenarios.

**Table 1:** **Distribution of low- to very high-abundant sequences detected in the four SuperSAGE libraries from the control (380 ppm CO**_**2**_**), CO**_**2 **_**(700 ppm CO**_**2**_**), CO**_**2 **_**plus drought and drought**

**Library**		**Control (380 ppm)**	**CO**_**2 **_**(700 ppm)**	**CO**_**2 **_**+ drought**	**Drought**
# detected sequences		69,150	182,736	139,987	106,410
**Abundance classes of detected sequences**					
# very high-abundant:	> 5000 tpm	37 (0.05%)	31 (0.02%)	34 (0.02%)	34 (0.03%)
# high-abundant:	500 – 5000 tpm	239 (0.35%)	252 (0.14%)	270 (0.19%)	263 (0.25%)
# mid-abundant:	50 – 500 tpm	863 (1.25%)	1,092 (0.60%)	1,126 (0.81%)	1,005 (0.95%)
# low-abundant:	< 50 tpm	68,013 (98.36%)	181,361 (99.25%)	138,557 (98.98%)	105,108 (98.78%)

Total amount of detected sequences per treatment are indicated and %-values are related to these total number of sequences.

**Table 2:** **Distribution of low-abundant sequences found uniquely under control conditions (380 ppm CO**_**2**_**), under elevated CO**_**2 **_**(700 ppm CO**_**2**_**), under elevated CO**_**2 **_**plus drought and drought (380 ppm CO**_**2**_**), or found to be common in all four SuperSAGE libraries at one time**

**Library**	**Control (380 ppm)**	**CO**_**2 **_**(700 ppm)**	**CO**_**2 **_**+ drought**	**Drought (380 ppm)**	**All libraries**
**# low abundant unique tags**					
**Original dataset**	4,260 (99.00%)	40,685 (99.76%)	15,865 (99.69%)	8,480 (99.12%)	24,131 (94.55%)
**tpm >0.8**	1,366 (96.95%)	1,438 (93.68%)	1,013 (95.39%)	1,168 (93.97%)	16,036 (91.86%)
**tpm >0.8; score >40; w/o “no hits”**	429 (96.84%)	374 (88.84%)	254 (94.07%)	334 (94.89%)	3,207 (90.16%)

Data were analyzed for different filter criteria.

We also performed a pairwise comparison of the libraries according to possible global change scenarios: control *vs*. drought (AmK *vs.* AmT), control *vs*. elevated CO_2_ (AmK *vs.* AmC) and control *vs*. CO_2_ + drought (AmK *vs*. AmCT). Using the STDGE2GO-Tool kit, we first searched the AmK *vs*. the AmT library for the following parameters: *Ambrosia*, ragweed, pollen, extensin, exine, intine, cell wall, coat and allergen. Using the term *Ambrosia*, 50 differentially expressed genes were identified that were mainly related to an *Ambrosia trifida* pollen cDNA library. All of the genes with a clear homologue and not only the description pollen cDNA library are listed in Table 3. The term ragweed resulted in 48 differentially expressed genes that were also found in the *Ambrosia* search. The search term “pollen” showed 48 hits that were primarily related to an *Ambrosia trifida* pollen cDNA library and thus also present in the *Ambrosia* search. For pollen, we also carried out a search for exine, intine, extensin, coat and cell wall. However, no additional hits were found. Searching for “allergen” identified 4 *Ambrosia* genes, a calcium-binding protein isoallergen 1, Amb a 1.1, Amb a 1.2 and Amb a 1.3 that were all up-regulated under drought stress (Table 3). In total, we could identify eight transcripts for allergenic proteins from *A. artemisiifolia*: two calcium-binding proteins (EF hand domain, Amb a 9 and Amb a 10), pectate lyases (Amb a 1.1, Amb a 1.2, Amb a 1.3 and Amb a 1.2 precursor), an actin-binding protein (profilin-like) and a cystatin proteinase inhibitor (Amb a CPI) (shown bold in Table 3). Except for the transcript of the Amb a 1.2 precursor protein, all of the other transcripts were up-regulated under drought. However, four of these transcripts were below the threshold of 1.5-fold (log_2_ = 0.59). The transcript for a homologue of a down-regulated ABA-responsive HVA22 protein from *A. trifida* was found in very high abundance (more than 10,000 tpm). In vegetative tissue, the *HVA22* genes are expressed in different tissues and show high levels of expression in flowers and inflorescences [79]. Drought stress suppressed *HVA22a* and *HVA22c* expression, had little effect on *HVA22e* expression and enhanced *HVA22d* expression in the inflorescent stems of *Arabidopsis*[79]. No changes or only small effects could be observed in the flower buds, except for a slight enhancement of expression under drought stress [79]. In accordance with our results, this result indicates that in addition to stress, the *HVA22* genes may also be important for the reproduction of plants. A homologue for a putative pollen-specific transcript from *A. trifida* was also found in high abundance and was down-regulated by drought. Other pollen-specific sequences were homologues to a pistil- and pollen-expressed gene from sunflower (*SF21*), a pollen coat protein transcript from wild cabbage and a pollen-specific actin-depolymerising factor from tobacco that were both down-regulated. *SF21* belongs to a gene family expressed in pollen and pistil in angiosperms and the encoded protein is important for pollen-pistil interactions [80,81]; however, the molecular function is still unknown. A search with the term “drought” resulted in 38 transcripts to homologues of a drought-stress subtracted cDNA library of safflower, also belonging to the Asteraceae and 35 of these cDNAs were up-regulated in drought-stressed ragweed pollen. Among these cDNAs, homologues to a carbonic anhydrase 3, a cyclophilin and a plastocyanin, proteins that are known to be allergenic, showed highly up-regulated transcripts (AllFam database; (http://www.meduniwien.ac.at/allergens/allfam/chart.php?kingdom=Plants&exposure=Inhalation&list=10&page=0). Interestingly, a highly up-regulated transcript for a CBS (cystathionine β-synthase) domain-containing protein homologue of *A. trifida* was detected (log_2_ = 9.01). CBS domain-containing proteins can sense cell energy levels and regulate redox homeostasis [82,83]. These proteins are important for stress regulation and corresponding genes are up-regulated upon drought stress [84].

**Table 3:** Up- and down-regulated transcripts in pollen of ragweed from control and drought stressed plants

**Database-id**	**Database**	**Description**	**Normalized tags per million**		**p-value**	**Fold change (log**_**2**_**)**
			**380 ppm CO**_**2**_	**380 ppm CO**_**2 **_**+ drought**		
TC52169	Asteraceae_TIGR	*A. artemisiifolia*, calcium-binding, **pollen allergen Amb a 9.1**	25*.*43	165*.*44	0	+2.70
296281908	Asteraceae	*A. trifida*, putative 60S ribosomal protein L34	685*.*5	1863*.*05	0	+1.44
255779233	Asteraceae	*A. trifida*, ß-glucosidase	196*.*37	391*.*4	0	+1.00
255779153	Asteraceae	*A. trifida*, SF16 protein	796*.*31	1293*.*52	0	+0.70
296281890	Asteraceae	*A. trifida*, conserved hypothetical protein	1463*.*21	2371*.*92	0	+0.70
302127809	GDB	*A. artemisiifolia*, **pollen allergen Amb a 1.1**	615*.*23	852*.*94	0	+0.47
296281926	Asteraceae	*A. trifida*, putative pollen-specific protein	8292*.*9	4704*.*67	0	−0*.*82
166438	GDB	*A. artemisiifolia*, **Amb a 1.2 precursor protein**	372*.*17	184*.*79	0	−1*.*01
255779319	Asteraceae	*A. trifida*, abscisic acid-responsive HVA22 family protein	22129*.*39	10765*.*07	0	−1*.*04
255779264	Asteracea	*A. trifida*, hypothetical protein	433*.*43	159*.*72	0	−1*.*44
283962764	Asteraceae	*A. trifida*, conserved hypothetical protein	410*.*87	96*.*73	0	−2*.*09
296281756	Asteraceae	*A. trifida*, putative ribokinase	71*.*21	0*.*05	0	−10*.*48
296281901	Asteraceae	*A. trifida*, putative CBS domain-containing protein	0*.*05	25*.*69	6.64e-39	+9.01
296281858	Asteraceae	*A. trifida*, unnamed protein product	18*.*8	62*.*53	1.70e-29	+1.73
296281775	Asteraceae	*A. trifida*, putative golgin-84-like protein	44*.*45	10*.*68	1.53e-28	−2*.*06
62249490	GDB	*A. artemisiifolia*, calcium-binding, **pollen allergen Amb a 10**	82*.*71	155*.*07	3.32e-27	+0.91
190607111	GDB	*A. trifida*, gibberellin-regulated protein	24*.*99	2*.*79	1.27e-26	−3*.*17
255779170	Asteraceae	*A. trifida*, photosystem I reaction center subunit K	1*.*11	18*.*42	1.32e-21	+4.06
255779131	Asteraceae	*A. trifida*, amino acid transporter	409*.*32	300*.*55	2.52e-21	−0*.*45
437311	Asteraceae	*A. artemisiifolia*, **cystatin proteinase inhibitor**	1459*.*28	1657*.*06	3.42e-16	+0.18
296281781	Asteraceae	*A. trifida* putative epoxide hydrolase	0*.*05	9*.*13	2.99e-14	+7.51
302127815	GDB	*A. artemisiifolia*, **pollen allergen Amb a 1.3**	113*.*89	168*.*07	1.21e-13	+0.56
296281905	Asteraceae	*A. trifida*, clathrin assembly protein	1025*.*19	896*.*55	1.15e-11	−0*.*19
296281843	Asteraceae	*A. trifida*, unnamed protein product	1.99	11.61	1.03e-09	+2.54
296281917	Asteraceae	*A. trifida*, DNA-directed RNA polymerase family	0*.*89	8*.*82	1.53e-09	+3.32
296281822	Asteraceae	*A. trifida*, putative signal peptidase	1*.*55	8*.*82	1.45e-07	+2.51
255779252	Asteraceae	*A. trifida*, 60S ribosomal protein L38	1*.*77	9*.*13	2.23e-07	+2.37
302127811	GDB	*A. artemisiifolia*, **pollen allergen Amb a 1.2**	30*.*08	47*.*36	6.73e-06	+0.66
34851181	GDB	*A. artemisiifolia*, **profilin-like protein (D03)**	40*.*25	58*.*81	1.90e-05	+0.55
TC40290	Asteraceae_TIGR	Pollen-specific protein SF21 (*Helianthus annuus*)	11*.*28	508*.*09	0	+5.49
TC52779	Asteraceae_TIGR	Pollen-coat protein (*Brassica oleracea*)	710*.*51	80167	0	−3*.*15
DY921400	Asteraceae_TIGR	Pollen-specific actin-depolymerizing factor 2 (*Nicotiana tabac*.)	21*.*23	7*.*12	2.24e-10	−1*.*58
DC239985	Asteraceae_TIGR	Profilin-6 (*Hevea brasiliensis*)	0*.*66	33*.*12	7.01e-45	+5.64
TC8863	Asteraceae_TIGR	α-Expansin precursor (*Nicotiana tabacum*)	1*.*77	17*.*02	3.82e-17	+3.27
33323054	GDB	Acidic chitinase (*Ficus awkeotsang*)	0*.*05	46*.*89	0	+9.87
261291803	Asteraceae	Cyclophilin (*Carthamus tinctorius*)	0*.*05	18*.*11	1.39e-27	+8.50
195607463	GDB	Aspartic proteinase nepenthesin-2 precursor (*Zea mays*)	24*.*33	0*.*05	3.32e-43	−8*.*93
28959515	Asteraceae	Carbonic anhydrase 3 (*Carthamus tinctorius*)	0*.*05	194*.*23	0	+11.92
FS486814	Asteraceae_TIGR	2-Cys peroxiredoxin-like protein (*Arabidopsis thaliana*)	0*.*66	17*.*02	9.27e-22	+4.68
289595531	Asteraceae	Plastocyanin (*Carthamus tinctorius*)	0*.*05	5*.*42	1.01e-08	+6.76
TC5518	Asteraceae_TIGR	Pathogenesis-related protein 5–1 (*Helianthus annuus*)	7*.*96	29*.*87	1.31e-16	+1.91

Plants were grown in the greenhouse under control (380 ppm CO_2_) and drought stress (380 ppm CO_2_ + drought). Using the STDGE-tool kit from GenXPro data were filtered for the terms *Ambrosia*, ragweed, pollen, extensin, exine, intine, cell wall, coat, allergen and the Allfam database. Known allergenic proteins in *Ambrosia* are shown in bold.

Next, we searched the AllFam database of allergen families, restricted to plants and inhalation. This search included 59 allergen families with 233 allergens (http://www.meduniwien.ac.at/allergens/allfam/chart.php?kingdom=Plants&exposure=Inhalation&list=10&page=0). In this search, the p-value was set to < E^−10^, except for safflower, which belongs also to the Asteraceae. In addition to the known allergens found under the *Ambrosia* search, eight transcripts for putative allergenic proteins from other plants according to the Allfam database were identified (Table 3). Seven of these transcripts were clearly up-regulated under drought, at least by a three-fold log_2_ change. In contrast, a homologue to an aspartic proteinase precursor from maize was down-regulated. The highest abundances were seen for the transcripts homologous to a profilin of rubber tree, an acidic chitinase of jelly fig and a safflower carbonic anhydrase. As pathogenesis-related (PR) proteins are known to be allergenic, we also searched for this term, coming up with a single hit for PR 5–1 homologue of sunflower, which was up-regulated under drought (Table 3). However, it is important to note that the abundances of all of these transcripts are low as compared to the ‘Amb a’ abundances in ragweed pollen.

The search of the AmK *vs*. the AmC library was performed for the terms given above. Under elevated CO_2_ concentration, the term *Ambrosia* resulted in 62 differentially regulated transcripts that were also mainly related to an *A. trifida* pollen cDNA library and the specified homologues are given in Tables 4 and 5. A search for ragweed resulted in 57 transcripts that were already present in the *Ambrosia* search. Under the search for allergen, five genes of *A. artemisiifolia* were identified: Amb a 1.1, Amb a 1.2, Amb a 1.3 and calcium-binding protein isoallergen 1 were up-regulated under elevated CO_2_, while the low-abundant profilin isoallergen 1 was down-regulated (Tables 4 and 5). This increase in Amb a 1 transcripts is in accordance with an increased level of Amb a 1 protein content in ragweed pollen grown under increased CO_2_ concentrations [22]. In total, nine transcripts for allergenic proteins from *A. artemisiifolia* were identified: two calcium-binding proteins (Amb a 9, Amb a 10), pectate lyases (Amb a 1.1, Amb a 1.2, Amb a 1.3 and Amb a 1.2 precursor protein), a cystatin proteinase inhibitor (Amb a CPI), a profilin allergen (Amb a 8.1) (shown bold in Tables 4 and 5). Seven of these transcripts were up-regulated and two were down-regulated (Amb a CPI and Amb a 8.1) under elevated CO_2_. However, for two transcripts, the log_2_ fold change was below the threshold (Amb a CPI and Amb a 1.2 precursor). Although at low abundance, the transcript homologous to a lipid transfer protein (LTP) from *A. trifida* was highly up-regulated (log_2_ = 9.2) under elevated CO_2_. LTPs are basic proteins that are abundant in higher plants [85]. These proteins belong to the prolamin superfamily and their role in allergenicity has been reviewed recently [86]. Similar to the drought library, the homologue for an abscisic acid-responsive *HVA22* transcript of *A. trifida* was found in high abundance and was down-regulated under elevated CO_2_ concentrations (Table 4). The transcript for the homologue of a putative pollen-specific protein from *A. trifida* was present in very high abundance and was slightly down-regulated under the elevated CO_2_ regime (Table 4). In contrast, the transcript for the pollen-specific protein SF21 homolog from sunflower was clearly up-regulated (Table 5). Other up-regulated pollen proteins included transcripts for a homologue of a pollen tube protein from tobacco and a pistil-specific extensin-like protein from safflower, while the transcript for a homologue of a pollen coat protein from wild cabbage was down-regulated. However, this value was below the threshold. Although not directly linked to pollen, the transcript for a homologue of a seed coat protein from rapeseed was extremely up-regulated (log_2_ = 12.99) (Table 5). The general search for pollen showed 59 transcripts and 56 out of these transcripts were from the pollen cDNA of *A. trifida*. Other highly regulated transcripts of *Ambrosia* included a ribokinase (log_2_ = −8.68) and a ribosomal protein L36 (log_2_ = 9.28).

**Table 4:** **Up- and down-regulated transcripts in pollen of ragweed from 380ppm CO**_**2 **_**(control) and 700ppm CO**_**2 **_**concentrations filtered for the terms *Ambrosia*, ragweed, pollen, extensin, exine, intine, cell wall, coat, allergen and the Allfam database**

**Database-id**	**Database**	**Description**	**Normalized tags per million**		**p-value**	**Fold change (log**_**2**_**)**
			**380 ppm CO**_**2**_	**700 ppm CO**_**2**_		
296281908	Asteraceae	*A. trifida*, putative ribosomal protein L34	685.524	0*.*05	0	−13*.*74
255779233	Asteraceae	*A. trifida*, ß-glucosidase	196*.*37	0*.*05	0	−11*.*94
296281756	Asteraceae	*A. trifida*, putative ribokinase	71*.*21	0*.*17	0	−8*.*68
255779271	Asteraceae	*A. trifida,* conserved hypothetical protein	410*.*97	115*.*33	0	−1*.*83
255779264	Asteraceae	*A. trifida,* hypothetical protein	433*.*43	249*.*61	0	−0*.*8
255779319	Asteraceae	*A. trifida,* abscisic acid-responsive HVA22 family	22129*.*3	13586*.*99	0	−0*.*7
296281926	Asteraceae	*A. trifida,* putative pollen-specific protein	8282*.*9	5596*.*37	0	−0*.*57
302127809	GDB	*A. artemisiifolia*, pectate lyase, **pollen allergen Amb a 1.1**	615*.*2	1102*.*65	0	+0.84
166442	GDB	*A. artemisiifolia,* pectate lyase**, pollen allergen Amb a 1.3**	113*.*89	233*.*1	0	+1.03
62249490	GDB	*A. artemisiifolia,* calcium binding, **pollen allergen Amb a 10**	82*.*71	218*.*2	0	+1.40
TC52169	Asteraceae_TIGR	*A. artemisiifolia,* calcium binding*,***pollen allergen Amb a 9.1**	25*.*43	136*.*31	0	+2.42
255779240	Asteraceae	*A. trifida*, lipid transfer protein	0*.*05	29*.*38	0	+9.20
296281913	Asteraceae	*A. trifida*, putative 60S ribosomal protein L36	0*.*22	137*.*41	0	+9.28
296281836	Asteraceae	*A. trifida,* putative o-linked n-acetylglucosamine transferase	1*.*77	25*.*44	9.34e-34	+3.85
302127811	GDB	*A. artemisiifolia*, pectate lyase, **pollen allergen Amb a 1.2**	30*.*08	71*.*62	6.40e-27	+1.25
437311	GDB	*A. artemisiifolia,***cystatin proteinase inhibitor**	1459*.*28	1258*.*73	2.95e-25	−0*.*21
255779252	Asteraceae	*A. trifida,* 60S ribosomal protein L38	1*.*77	17*.*16	1.24e-20	+3.28
296281775	Asteraceae	*A. trifida,* putative golgin-84-like protein	44*.*45	19*.*82	3.03e-18	−1*.*66
34851181	GDB	*A. artemisiifolia,***profilin-like protein (D03)**	40*.*25	74*.*76	4.07e-17	+0.89
255779129	Asteraceae	*A. trifida,* 60S ribosomal protein	1*.*99	13*.*56	2.14e-14	+2.77
190607080	GDB	*A. trifida,* putative galactan: galactan galactosyltransferase	303*.*84	378*.*62	3.49e-14	+0.32
166438	GDB	*A. artemisiifolia*, pectate lyase, **Amb a 1.2 precursor protein**	372*.*17	451*.*88	1.85e-13	+0.28
296281810	Asteraceae	*A. trifida*, putative ribosomal protein L5	2*.*65	13*.*91	7.56e-13	+2.39
255779131	Asteraceae	*A. trifida*, amino acid transporter	409*.*32	337*.*7	1.27e-12	−0*.*28
296282845	Asteraceae	*A. trifida*, putative stellacyanin	25*.*87	49*.*49	1.30e-12	+0.94
296281843	Asteracea	*A. trifida,* unnamed protein product	0*.*89	9*.*22	7.99e-12	+3.38
190607111	Asteraceae	*A. trifida,* gibberellin-regulated protein	24*.*99	10*.*78	1*.*28E-11	−1*.*22

Plants were grown in the greenhouse under control (380 ppm CO_2_) and 700 ppm CO_2_ concentrations. Using the STDGE-tool kit from GenXPro data were filtered for the terms *Ambrosia*, ragweed, pollen, extensin, exine, intine, cell wall, coat, allergen and the Allfam database. Known allergenic proteins in *Ambrosia* are shown in bold.

**Table 5:** **Up- and down-regulated transcripts in pollen of ragweed from 380ppm CO**_**2 **_**(control) and 700ppm CO**_**2 **_**concentrations filtered for the terms *Ambrosia*, ragweed, pollen, extensin, exine, intine, cell wall, coat, allergen and the Allfam database**

**Database-id**	**Database**	**Description**	**Normalized tags per million**		**p-value**	**Fold change (log**_**2**_**)**
			**380 ppm CO**_**2**_	**700 ppm CO**_**2**_		
296281905	Asteraceae	*A. trifida,* putative clathrin assembly protein	1025*.*19	1139*.*63	5.92e-11	+0.15
296281822	Asteraceae	*A. trifida,* putative signal peptidase	1.548	9*.*68	3.62e-10	+2.64
296281744	Asteraceae	*A. trifida,* conserved hypothetical protein	1*.*77	9*.*97	6.62e-10	+2.49
296281890	Asteraceae	*A. trifida,* conserved hypothetical protein	1463*.*71	1586*.*87	2.83e-09	+0.11
296281917	Asteraceae	*A. trifida,* DNA-directed RNA polymerase family protein	0*.*89	7*.*01	1.97e-08	+2.99
TC43769	Asteraceae_TIGR	*A. artemisiifolia*, profilin, **pollen allergen Amb a 8.1**	4*.*64	0*.*64	3.27e-08	−2*.*86
255779153	Asteraceae	*A. trifida,* SF26 protein	796*.*31	879*.*06	7.45e-08	+0.14
296281873	Asteraceae	*A. trifida,* putative mitochondrial ATP synthase 6 kDa subunit	9*.*29	20*.*23	1.94e-07	+1.12
255779194	Asteraceae	*A. trifida*, putative cullin-1-protein	0*.*44	4*.*87	9.01e-07	+3.46
296281858	Asteraceae	*A. trifida*, unnamed protein product	18*.*8	32*.*11	1.20e-06	+0.77
255777293	Asteraceae	*A. trifida,* mitochondrial outer membrane membrane protein	3*.*76	10*.*43	5*.*873-06	+1.37
296281875	Asteraceae	*A. trifida*, signal peptidase subunit family protein	0*.*05	2*.*9	1.40e-05	+5.87
296281777	Asetraceae	*A. trifida*, calmodulin-like protein	8*.*85	17*.*1	2.93e-05	+0.95
255779292	Asteraceae	*A. trifida*, 60S ribosomal protein L35a	1*.*33	0*.*05	3.33e-05	−4*.*73
255779177	Asteraceae	*A. trifida*, putative CREG1	1*.*33	0*.*05	3.33e-05	−4*.*73
TC40290	Asteraceae_TIGR	Pollen-specific protein SF21 (*Helianthus annuus*)	11*.*28	166*.*45	0	+3.88
TC52779	Asteraceae_TIGR	Pollen coat protein (*Brassica oleracea*)	710*.*51	539*.*79	2.22e-39	−0*.*4
TC5878	Asteraceae_TIGR	Pollen tube RhoGDI2 (*Nicotiana tabacum*)	0*.*05	9*.*97	6.50e-18	+7.64
261291923	Asteraceae	Pistil-specific extensin-like protein (*Carthamus tinctorius*)	0*.*89	4*.*93	2.31e-05	+2.47
126480015	GDB	Seed coat (*Brassica napus*)	0*.*22	1800*.*66	0	+12.99
33323054	GDB	Acidic chitinase (*Ficus awkeotsang*)	0*.*05	58*.*65	0	+10.20
TC7736	Asteraceae_TIGR	Carbonic anhydrase (*Solanum lycopersicum*)	0*.*05	9*.*39	6.67e-17	+7.55
FS486814	Asteraceae_TIGR	2-Cys peroxiredoxin-like protein (*Arabidopsis thaliana*)	0*.*66	114*.*17	0	+7.43
BU019358	Asteraceae_TIGR	Thioredoxin (*Medicago trunculata*)	305.83	600.70	0	+0.97
AI100454	All_TIGR_Plant.fa	Serine/threonine protein kinase (*Brassica napus*)	152.14	34.25	0	−2*.*15
195607463	GDB	Aspartic proteinase nephentesin precursor (*Zea mays*)	24.33	3.01	2.99e-38	−3.01
GR085079	Asteraceae	Lipid-transfer protein (*Salvia miltiorrhiza*)	0.05	17.21	1.28-23	+9.20
TC5118	Asteraceae_TIGR	Pathogenesis-related protein 5–1 (*Helianthus annus*)	7.96	33.21	1.16e-24	+2.06

Known *Ambrosia artemisiifolia* allergens are shown in bold.

The AllFam database search indicated seven transcripts for putative allergenic proteins from other plants. Five of these proteins were up-regulated under elevated CO_2_ concentrations, whereas the transcripts of a protein kinase and an aspartic proteinase were down-regulated, similar as under drought stress (Table 5). Interestingly, the transcript of a homologue for a non-specific lipid-transfer protein of red sage was also strongly up-regulated, although at low abundance. As described for the drought stress conditions, the transcript level of PR 5–1 homologue from sunflower was also elevated (Table 5).

In a final step, we compared the *Ambrosia* control library (AmK) *vs.* the elevated CO_2_ + drought-stressed library (AmCT). Under the search term *Ambrosia*, 55 transcripts and for ragweed 50 transcripts, mainly homologues from *A. trifida*, were identified. The homology description is given in Table 6. The search term “allergen” resulted in five trancripts from *A. artemisiifolia* and the calcium-binding protein isoallergen 1, Amb a 1.1, Amb a 1.2 and Amb a 1.3 were up-regulated (Table 6). In total, eight transcripts of up-regulated allergenic proteins were identified for *A. artemisiifolia*: two calcium-binding proteins (Amb a 9 and Amb a10), pectate lyases (Amb a 1.1, Amb a 1.2, Amb a 1.3 and Amb a 1.2 precursor), a profilin-like protein (Amb a 8) and a cystatin proteinase inhibitor (Amb a CPI). However, the change of Amb a 1.2 precursor and Amb a CPI were below the threshold of 1.5. An LTP homologue from *A. trifida* was highly up-regulated (Table 6). The transcript of a low-abundance aspartic protease homologue from *A. trifida*, allergenic according to the AllFam database, was highly up-regulated (Table 6). The transcript of the very high abundant pollen-specific protein homologue from *A. trifida* was slightly down-regulated, similar to the other two libraries, while the transcript of the pollen-specific protein SF21 homologue from sunflower was up-regulated (Table 6). The transcript of a pollen coat protein homologue from wild cabbage was slightly down-regulated and the seed coat protein transcripts homologous to the one from rapeseed was extremely highly up-regulated (log_2_ = 14.71) (Table 6). The general search for pollen resulted in 51 transcripts that were mainly related to the *A. trifida* pollen cDNA library. The search input drought resulted in 33 differentially regulated transcripts with homology to a safflower drought stress-subtracted library and 25 of these transcripts were up-regulated. The homologue of an ABA-responsive HVA22 transcript from *A. trifida* was down-regulated, as in the other two libraries. Although at low abundance, the transcript for the CBS domain-containing protein was highly up-regulated (Table 6).

**Table 6:** **Up- and down-regulated transcripts in pollen of ragweed plants grown under control (380 ppm CO**_**2**_**) and 700 ppm CO**_**2 **_**+ drought conditions**

**Database-id**	**Database**	**Description**	**Normalized tags per million**		**p-value**	**Fold change (log**_**2**_**)**
			**380 ppm CO**_**2**_	**700 ppm CO**_**2 **_**+ drought**		
TC52169	Asteraceae	*A. artemisiifolia*, calcium-binding, **pollen allergen Amb a 9.1**	25*.*43	336	0	+3.72
296281845	Asteraceae	*A. trifida*, putative stellacyanin	28*.*87	94*.*64	0	+1.87
62249490	GDB	*A. artemisiifolia*, calcium-binding, **pollen allergen Amb a 10**	82*.*71	276*.*12	0	+1.74
302127811	GDB	*A. artemisiifolia*, **pollen allergen Amb a 1.2**	30*.*08	98*.*3	0	+1.71
302127809	GDB	*A. artemisiifolia*, **pollen allergen Amb a 1.1**	615*.*21	1817*.*41	0	+1.56
302127821	GDB	*A. artemisiifolia*, **pollen allergen Amb a 1.3**	113*.*89	262*.*9	0	+1.21
190607080	GDB	*A. trifida*, putative galactan: galactan galactosyltransferase	303*.*84	657*.*37	0	+1.11
296281908	Asteraceae	*A. trifida*, putative 60S ribosomal protein L34	685*.*52	1476*.*56	0	+1.11
296281890	Asteraceae	*A. trifida*, conserved hypothetical protein	1463*.*71	2258*.*91	0	+0.63
296281905	Asteraceae	*A. trifida*, putative clathrin assembly protein	1025*.*19	1528*.*93	0	+0.58
296281926	Asteraceae	*A. trifida*, putative pollen-specific protein	8282*.*9	5812*.*3	0	−0*.*51
255779264	Asteraceae	*A. trifida*, hypothetical protein	433.428	211*.*61	0	−1*.*03
255779319	Asteraceae	*A. trifida*, abscisic acid-responsive HVA22 family protein	22129*.*39	8957*.*98	0	−1*.*3
255779233	Asteraceae	*A. trifida*, ß-glucosidase	196*.*37	0*.*05	0	−7*.*5
296281756	Asteraceae	*A. trifida*, putative ribokinase	71*.*21	0*.*1	0	−9*.*49
255779271	Asteraceae	*A. trifida*, conserved hypothetical protein	410*.*87	0*.*05	0	−13
255779240	Asteraceae	*A. trifida*, lipid transfer protein	0*.*05	27*.*37	5.61e-45	+9.10
34851181	GDB	*A. artemisiifolia*, **profilin-like protein (D03)**	40*.*25	103*.*93	3.17e-39	+1.37
296281775	Asteraceae	*A. trifida*, golgin-84-like protein	44*.*45	9*.*68	4.05e-38	−2*.*2
255779129	Asteraceae	*A. trifida*, 60S ribosomal protein	1*.*99	30*.*13	3.40e-37	+3.93
296281830	Asteraceae	*A. trifida*, aspartic protease	0*.*44	15*.*51	1.13e-22	+5.13
296281858	Asteraceae	*A. trifida*, unnamed protein product	18*.*8	51*.*87	2.81e-22	+1.46
437311	GDB	*A. artemisiifolia*, **cystatin proteinase inhibitor**	1459*.*28	1651*.*63	8*.*89E-18	+0.18
296281901	Asteraceae	*A. trifida*, CBS domain-containing protein	0*.*05	9*.*68	2.64e-14	+7.60
296281817	Asteraceae	*A. trifida*, unnamed protein	0*.*05	8*.*1	9.73e-14	+7.34
296281822	Asteraceae	*A. trifida*, signal peptidase	1.55	11.36	1.08e-11	+2.88
255779271	Asteraceae	*A. trifida*, conserved hypothetical protein	0*.*05	6*.*03	2.27e-10	+6.90
255779252	Asteraceae	*A. trifida*, 60S ribosomal protein L34	0*.*22	4*.*45	1.27e-06	+4.33
255779153	Asteraceae	*A. trifida*, SF16 protein	796*.*31	875*.*21	1.54e.06	+0.14
296281737	Asteraceae	*A. trifida*, SKIP interacting protein	3*.*1	10*.*27	1.71e-06	+1.73
255779293	Asteraceae	*A. trifida*, mitochondrial outer membrane protein porin	3*.*76	10*.*87	6.08e-06	+1.53
296281875	Asteraceae	*A. trifida*, signal peptidase subunit family protein	0*.*05	3*.*26	7.04e-06	+6.03
255779131	Asteraceae	*A. trifida*, amino acid transporter	0*.*89	5*.*34	1.10e-05	−0*.*18
166438	GDB	*A. artemisiifolia*, **Amb a 1.2 precursor protein**	372*.*17	420*.*86	1.71e-05	+0.18
TC40290	Asteraceae	Pollen-specific protein SF21 (*Helianthus annuus*)	11*.*28	678*.*11	0	+5.91
TC52779	Asteraceae	Pollen coat protein (*Brassica oleracera*)	710*.*51	464*.*53	2.74e-14	−0*.*61
1268001	GDB	Seed coat (*Brassica napus*)	0*.*22	5926*.*21	0	+14.71
DC239985	Asteraceae	Profilin-6 (*Hevea brasiliensis*)	0.663	20*.*06	1.68e-28	+4.92
195607463	GDB	Aspartic proteinase nephentesin-2 precursor (*Zea mays*)	24*.*33	0*.*05	0	−8*.*93
FS486814	Asteraceae	2-Cys peroxiredoxin-like protein (*Arabidopsis thaliana*)	0.663	94*.*25	0	+7.15
TC51674	Asteraceae	Thioredoxin h (*Pisum sativum*)	5*.*09	21*.*14	9.27e-15	+2.06
242346662	GDB	Kunitz-type protease inhibitor (*Populus trichocarpa x P. nigra*)	23*.*44	8*.*24	1.80e-20	−2*.*16

Plants were grown in the greenhouse under control (380 ppm CO_2_) and 700 ppm CO_2_ + drought. Using the STDGE-tool kit from GenXPro data were filtered for the terms *Ambrosia*, ragweed, pollen, extensin, exine, intine, cell wall, coat, allergen and the Allfam database. Known allergenic proteins in *Ambrosia* are shown in bold.

The AllFam database indicated five additional transcripts for allergenic proteins. Three of these transcripts were up-regulated and two were down-regulated (Table 6).

### Quantitative real-time RT-PCR (qRT-PCR)

**Figure 4:**
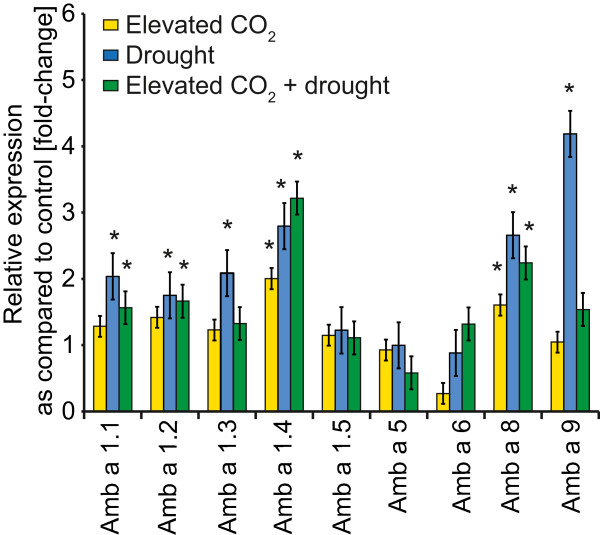
**Quantitative real-time RT-PCR of selected ragweed allergens.** The relative expression is indicated as fold change. The gene-specific primers are given in Additional file 8. As a reference gene, α-tubulin was used. The bars indicate SE and an asterisk indicates significant changes; N = 4 individual plants and three technical replicates.

qRT-PCR was performed for selected ‘Amb a’ transcripts (Figure 4). The relative expression rate ranged from 1 to 4 and increased for Amb a 1.1, Amb a 1.2, Amb a 1.3, Amb a 1.4, Amb a 8 and Amb a 9, while the expression levels of Amb a 1.5, Amb a 5 and Amb a 6 were not influenced or even reduced. The highest values were observed for drought and CO_2_ + drought (Figure 4) and Amb a 1.4, Amb a 8 and Amb a 9 showed the strongest increase. To validate the results from the SuperSAGE, we compared the log_2_ fold change of ‘Amb a’ transcripts found in the SuperSAGE libraries and the qRT-PCR results. For the Amb a 1 transcripts, a relatively good correlation was found. The best correlation was observed for the drought treatment, whereas the elevated CO_2_ and elevated CO_2_ + drought showed the same expression trend but not identical absolute values. Using only the significantly changed qRT-PCR ratios a significant correlation with the SuperSAGE data sets was found (Additional file 7). For Amb a 8, the qRT-PCR data contrasted the SuperSAGE data and for Amb a 9, the fold changes were much higher for the SuperSAGE data compared to the qRT-PCR values. However, this kind of result has also been reported in the literature with coincident and contrasting data for SuperSAGE and microarrays [87], as well as for the SuperSAGE and qRT-PCR analyses [88]. In sheepgrass differences up to a factor of 2.5 between digital gene expression data and RT-PCR ratio and even inconsistencies were reported [89]. In poplar differences by factors of 4–16 between microarray and qRT-PCR data were reported and in switchgrass also factors up to 15 were found [90,91]. This result reflects a general problem when comparing transcript abundance with different platforms and might be caused by allele-specific gene expression [88,92]. Moreover, it is interesting to note that transcript abundances are important when comparing different platforms and that good correlations were found for high abundance transcripts and a correlation decrease for lower abundance transcripts [93], as it was also given for Amb a 8 in this study.

## Conclusions

Our data on ragweed plants fumigated with elevated CO_2_ and drought stress conditions support the idea that pollen transcripts related to allergenicity are influenced by such global climate change factors. A strong up-regulation of ‘Amb a’ transcripts was evident under elevated CO_2_, drought stress and elevated CO_2_ + drought stress conditions. Based on normalized tags, Amb a 1.1 and Amb a CPI were expressed at the highest levels. The increased Amb a 1 transcript level is in accordance with an increased Amb a 1 protein content under elevated CO_2_ concentrations [22].This result clearly indicates that under expected global change conditions, the allergenicity of ragweed pollen may increase, thereby affecting human health. However, we cannot exclude the possibility that the increased ‘Amb a’ transcript level will also reflect the corresponding allergenic protein level, as an incongruent expression between transcripts and proteins is well described in the literature [94-96]. In addition to the well-known ‘Amb a’ transcripts, transcript homologies to other plant allergens were found that might modulate the ‘Amb a’ allergenic response. However, this possibility requires to be tested in suitable model systems.

## Methods

### Plant growth conditions

Ragweed seeds were collected from a single plant from an outdoor stand to avoid parental environmental effects on the growth and development of the next generation [97]. The experiment began on March 29, 2010. The plants were grown in fully air-conditioned greenhouse cabins, each 36 m^2^ (http://www.helmholtz-muenchen.de/en/eus/facilities/greenhouse/index.html) as recently described [21]. One cabin was fumigated with 380 ppm CO_2_ (control samples) and in the second the CO_2_ was enriched to 700 ppm (CO_2_ samples). The light conditions and temperatures were according to the outside (10°C - 35°C) and the relative humidity ranged from 55% -70% (Additional file 8). The watering of plants was carried automatically by a tube system applying 100 ml per pot each day. The drought stress began on May 21 by reducing the watering to 100 ml per 36 h. The pollen was collected continuously from August 9 to November 22 using a modified ARACON system (BETATECH, Ghent, Belgium) [17] and stored at −80°C until use.

### Pollen viability

The pollen viability was analyzed by the p-phenylenediamine test according to Rodriguez-Riano and Dafni [98].

### Analyses of phenolic metabolites

15 mg of frozen pollen was extracted with 1.2 ml phosphate buffer saline (PBS) for 1 h at room temperature (RT). After centrifugation the residue was then extracted with 1.2 ml MeOH for 1 h at RT. Reverse-phase high-performance liquid chromatography (RP-HPLC) separation of the aqueous and methanol extracts was as described by Ghirardo et al. [99].

### SuperSAGE libraries

Pollen from three single plants of each treatment were combined for RNA isolation. The isolation was carried out by GenXPro GmbH (Frankfurt, Germany) using the InviTrap® Spin Plant RNA Mini Kit (STRATEC Molecular GmbH, Berlin, Germany). In detail: 20–30 mg pollen was added to 900 μl lysis solution DCT + 10 μl 2-mercaptoethanol and homogenized for 2× 1 min at 30 Hz using a TissueLyser II by Retsch (QIAGEN, Hilden, Germany). The homogenate was then thoroughly mixed by vortexing and incubated for 10 min under continuous shaking. The remaining steps followed the kit instructions. The yield was 10–24 μg of total RNA (measured with Implen NanoPhotometer™ (Implen GmbH, München, Germany) using the LabelGuard™ Microliter Cell with LF10 lid. A DNAse I digestion was carried out with Baseline-ZERO DNAse (Biozym Scientific GmbH, Hessisch Oldendorf, Germany) in order to exclude even traces of genomic DNA. Purification of total RNA after DNAse I digestion was carried out with MACHEREY-NAGEL “NucleoSpinRNA Clean-up XS-Kit (MACHEREY-NAGEL, Düren, Germany). The quality of total RNA was checked on a Bioanalyzer with a 2100 expert Plant RNA Nano chip (Agilent Technologies, Waldbronn, Germany). The total RNA had RIN-values between 6.2 and 8.0.

The construction of the ST-DGE/SuperSAGE libraries was carried out by GenXPro essentially as described by Matsamura et al. [100] with the implementation of GenXPro-specific technology. For each of the 4 SuperSAGE libraries 5 μg of total RNA was applied for processing the ST-DGE library preparation with improved SOPs for quality control as well as specific bias proved adapters (patent pending) for elimination of PCR artifacts (TrueQuant methodology).

### Bioinformatic analysis

The four libraries L1 = AmK (380 ppm CO_2_), L2 = AmC (700 ppm CO_2_), L3 = AmCT (700 ppm CO_2_ + drought stress) and L4 = AmT (380 ppm CO_2_ + drought stress) were BLASTed against the Asteraceae databases of TIGR and NCBI and then against TIGR all plant and against the plant GDB. The pairwise comparison of the libraries was performed using the STDGE2GO-Tool analyses tool for gene ontology (GenXPro) with a score value of at least 36. For the probability of a tag to be differentially expressed, we used a p-value of < 0.0001 for Asteraceae and a p-value < E^−10^ for all other plants and a fold change of at least 1.5 [101]. The normalized values of each tag in relation to one million tags are listed (tpm = tags per million). Tags that are present zero times are replaced by 0.05 to allow for the calculation. According to the cumulative frequency distribution and approximately 40% - 50% of the expressed genes, a tpm threshold of > 0.8 was used for each of the library comparisons (Additional file 4) [72]. Additionally, MapMan [73] was used to group the SuperSAGE tags into distinct functional categories (BIN-codes). For this grouping, the SuperSAGE tags were first matched to *Ambrosia* 454-transcriptome data (contigs + singletons) by Kanter et al. [17], allowing a maximum of one mapping error per 26 mer. To define homologous *Arabidopsis* genes, the sequences of the *Ambrosia* (454-transcriptome) were compared to the gene set of *Arabidopsis* (TAIR10). For this comparison, a BLAST search was performed and the first best matched *Arabidopsis* gene was extracted. Furthermore, only first best hits with ≥ 70% identity covering at least 30 amino acids were assigned to each contig (workflow: Additional file 5). A total of 2,184 non-redundant *Arabidopsis* genes could be assigned to 454 contigs using SuperSAGE evidence. Next, the hit counts were calculated for each contig and to allow for a between-sample comparison, the hit counts were normalized and the tpm values were calculated. Moreover, for a pairwise comparison, the log_2_ fold-change (contig x, sample s_1_, control s_2_) = log_2_ [tpm (x,s_1_) / tpm (x,s_2_)] was calculated. For samples that were present zero times, the tpm was replaced by 0.05 to allow for the calculation of the ratio. The data were then filtered tpm > 0.8 and were analyzed by MapMan.

### qRT-PCR

RNA was isolated according to Kanter et al. [17]. The DNA digestion was performed with RQ1 RNase-Free DNAse (Promega, Mannheim, Germany). The RNA yield and quality were determined by spectral photometry at 230, 260 and 280 nm. Only RNA with acceptable ratios of 260/280 (>2.0) and 260/230 (>2.0) was used and reversed transcribed. Reverse transcription was carried out using 2 μg total RNA and superscript II reverse transcriptase according to the manufacturer’s instructions (Invitrogen, Karlsruhe, Germany).

The obtained cDNA was diluted 1:20 and the qRT-PCR was performed in a 20-μl reaction mixture of SYBR Green ROX mix (12.5 μl) (Thermo Scientific QPCR), 5 μl cDNA and 1.25 μl forward and reverse primer each using the ABIPrism 7500 fast real-time PCR system (Applied Biosystems, Darmstadt, Germany). The PCR conditions were as follows: 1 cycle at 50°C for 2 min, 1 cycle at 95°C for 10 min, 40 cycles at 95°C for 15 s and 60°C for 1 min. As an internal standard, α-tubulin was used; the relative expression was calculated using the REST© software tool [102]. The gene-specific primers for α-tubulin and ragweed allergens are given in Additional file 9.

## Competing interests

The authors declare that they have no competing interests.

## Authors’ contribution

JD, HB, CTH, UF and DE performed and designed the experiments. AE, FZ, WH and UF performed the experiments. AE, WH, RH, MP, UF and DE analysed the data. JBW was responsible for the greenhouse cabins. UF and DE wrote the manuscript. All authors read and approved the final manuscript.

## Supplementary Material

Additional file 1 Viability of ragweed pollen. Click here for file

Additional file 2 RP-HPLC diagram of water-soluble and methanol-extractable metabolites. Click here for file

Additional file 3 **SuperSAGE libraries. **Number of sequenced tags and tag frequencies. Click here for file

Additional file 4 Cumulative frequency distribution TPM values. Click here for file

Additional file 5 **Workflow of the *Ambrosia *** **transcriptome analysis via MapMan.** Click here for file

Additional file 6 **Interesting BIN-names detected by MapMan.** BIN-codes, BIN-names, the *Arabidopsis* gene ID as well as a short description are given. Log_2_ fold changes for treatments as compared to the control are shown. *Arabidopsis* sequence matches were grouped according to their log_2_ fold change value. Only values of a log_2_ fold change of at least 1.5 were considered important; blue = up-regulation (log_2_ > 1.5), yellow = down-regulation (log_2_ < −1.5). Click here for file

Additional file 7 **Correlation of SuperSAGE data with qRT-PCR data.** 1–4: drought stress, 1: Amb a 1.1; 2: Amb a 1.2, 3: Amb a 1.3; 4: Amb a 9; 5–6: 700 ppm CO_2_ + drought, 5: Amb a 1.1; 6: Amb a 1.2. Click here for file

Additional file 8 **Greenhouse data.** Temperature, relative humidity and light conditions in the greenhouse during the vegetation period of ragweed are given. Click here for file

Additional file 9 Sequences of primers that were used for quantitative real-time RT-PCR (qRT-PCR). Click here for file
