# Supplementary material for: Ragweed (Ambrosia artemisiifolia) pollen allergenicity: SuperSAGE transcriptomic analysis upon elevated CO2 and drought stress
Source: BMC Plant Biol. 2014 Jun 27;14:176. doi: 10.1186/1471-2229-14-176 (PMC4084800; doi:10.1186/1471-2229-14-176)
Supplement: Additional file 1 — Viability of ragweed pollen. [file 1471-2229-14-176-S1.pdf]

Additional file 1. Viability of ragweed pollen. Plants were grown under the indicated conditions over the whole growing season. (N = 3 for each treatment samples; 5 mg pollen per sample, 2000-3000 pollen were counted; t-test).

CO_2	Min (%)	Max (%)	Mean (%) ± SD	SE
350 ppm (1)	35	57	46 ± 5.5	1.2
700 ppm (2)	19	68	41 ± 17.8	4.0
350 ppm + drought (3)	12	50	24 ± 13.1	2.9
700 ppm + drought (4)	22	41	30 ± 4.8	1.1

p-values: 2 vs 1: 0.166; 3 vs 1: 0.000; 4 vs 1: 0.00; 4 vs 3: 0.144
