# Supplementary material for: Ragweed (Ambrosia artemisiifolia) pollen allergenicity: SuperSAGE transcriptomic analysis upon elevated CO2 and drought stress
Source: BMC Plant Biol. 2014 Jun 27;14:176. doi: 10.1186/1471-2229-14-176 (PMC4084800; doi:10.1186/1471-2229-14-176)
Supplement: Additional file 2 — RP-HPLC diagram of water-soluble and methanol-extractable metabolites. [file 1471-2229-14-176-S2.pdf]

a)

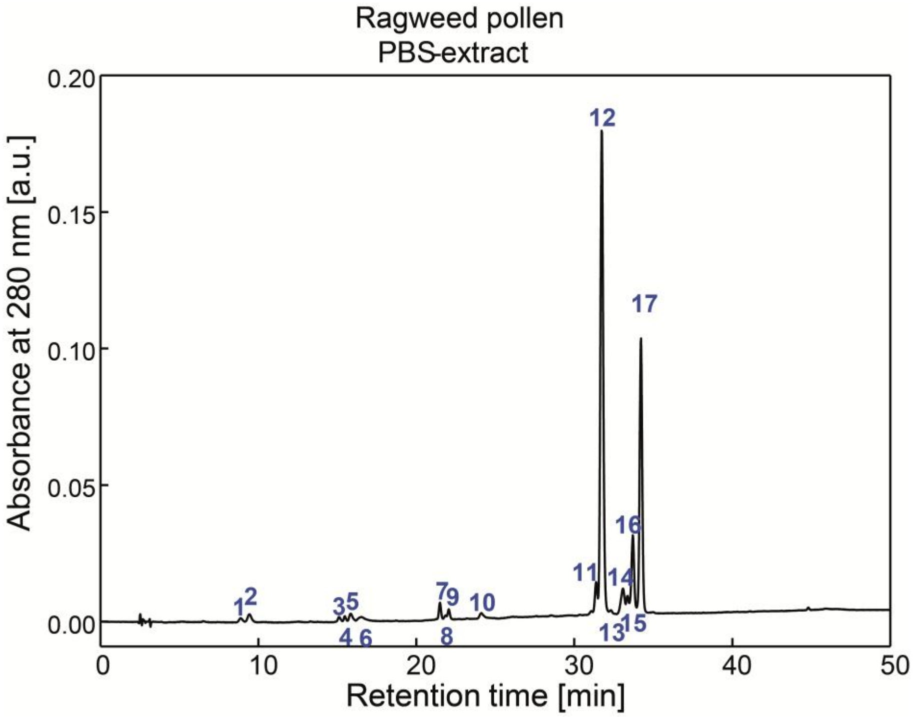

b)

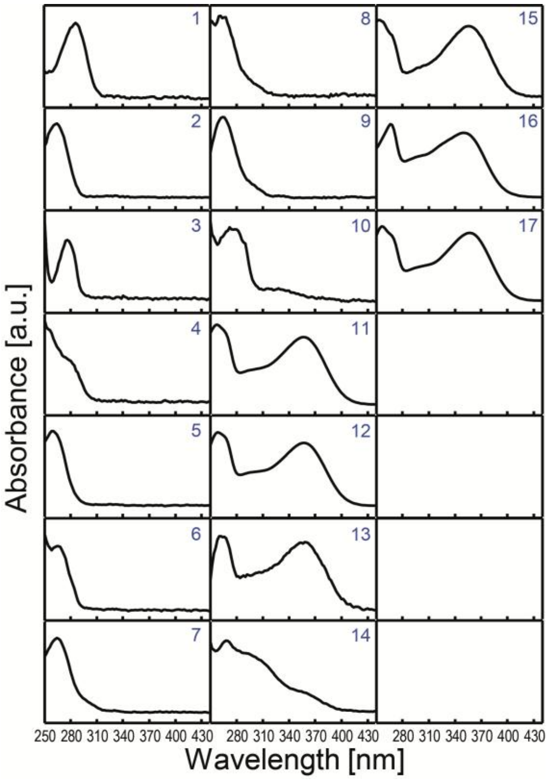

c)

#	Time	Maxima	Metabolite
1	8.8	285	
2	9.3	263	
3	14.8	274	
4	15.1	255-277	
5	15.4	258	
6	16.2	264	
7	21.4	263	
8	21.7	260-266	
9	22.0	263	
10	24.0	272-278-289	
11	31.4	256-356	Quercetin derivative
12	31.7	257-356	Quercetin derivative
13	32.3	261-264-356	Quercetin derivative
14	33.2	267	
15	33.4	252-266-354	Quercetin derivative
16	33.8	265-348	Kaempferol derivative
17	34.3	256-356	Quercetin derivative

d)

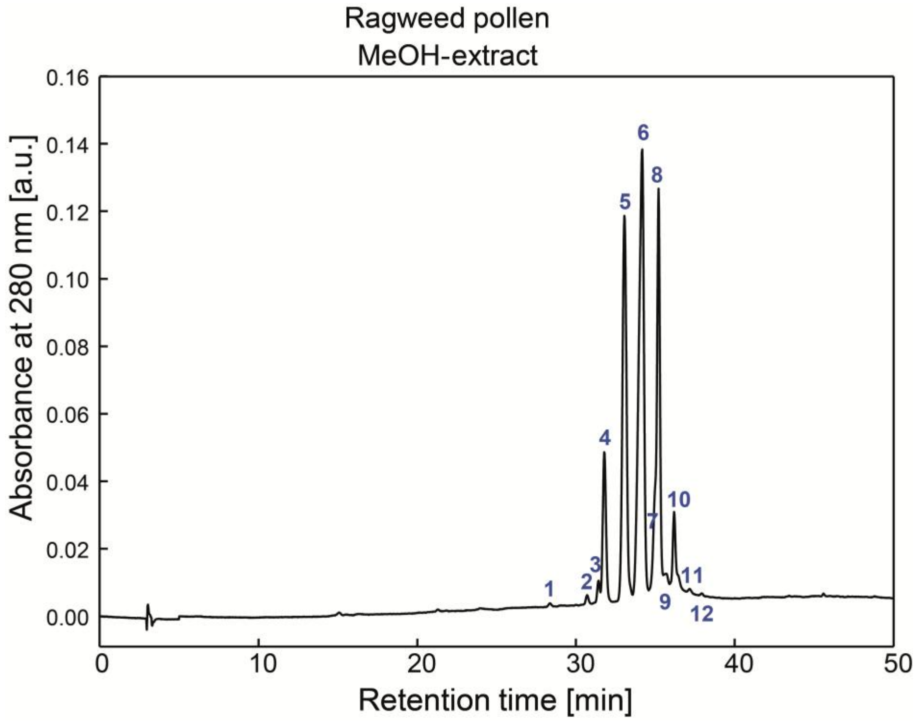

e)

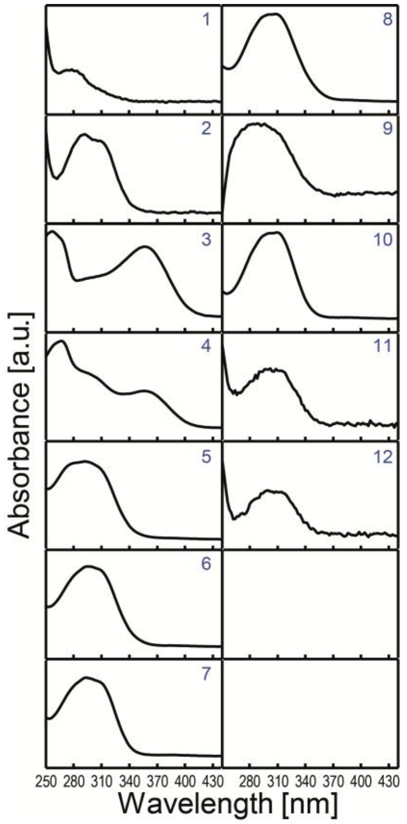

f)

#	Time	Maxima	Notice
1	28.4	276	
2	30.7	290-306	
3	31.4	255-355	= PBS 11
4	31.8	265-300-355	= PBS 12 + ?
5	33.1	290	
6	34.2	295	
7	35.0	292	
8	35.2	308	
9	35.6	290	
10	36.2	308	
11	37.1	308	
12	37.9	300-312	

Additional file 2. RP-HPLC diagrams of water soluble and methanol extractable metabolites.

a) shows a typical HPLC run of a PBS extract from *Ambrosia* pollen at an absorbance of 280 nm. **b)** gives the diode array spectra of the respective peeks between 250 and 430 nm. **c)** indicates the corresponding retention time and the peak maxima for each single peak stated in b); for some peaks preliminary structural assignments are given. **d)** gives a typical HPLC run of a methanol extract, following the PBS extraction, at an absorbance of 280 nm. **e)** shows diode array spectra and **f)** indicates corresponding retention times and peak maxima.
