# Supplementary material for: Ragweed (Ambrosia artemisiifolia) pollen allergenicity: SuperSAGE transcriptomic analysis upon elevated CO2 and drought stress
Source: BMC Plant Biol. 2014 Jun 27;14:176. doi: 10.1186/1471-2229-14-176 (PMC4084800; doi:10.1186/1471-2229-14-176)
Supplement: Additional file 5 — Workflow of the Ambrosia transcriptome analysis via MapMan. [file 1471-2229-14-176-S5.pdf]

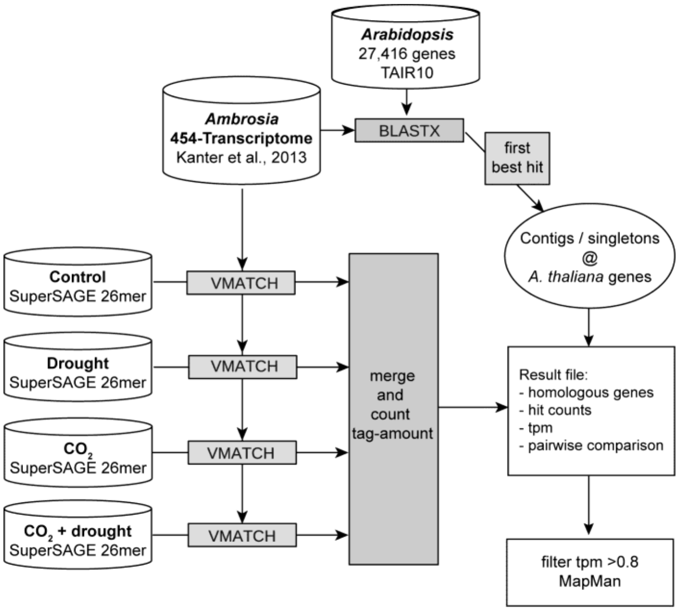

Additional file 5. Workflow for analyzing the SuperSAGE data via MapMan. SuperSAGE data were matched to *Ambrosia* transcriptome data [1] and BLASTed against *Arabidopsis*. *Arabidopsis* genes were then used for analysis in MapMan [2].

1. Kanter U, Heller W, Durner J, Winkler JB, Engel M, et al. (2013) Molecular and immunological characterization of ragweed (Ambrosia artemisiifolia L.) pollen after exposure of the plants to elevated ozone over a whole growing season. PLoS ONE 8: e61518.
2. Usadel B, Poree F, Nagel A, Lohse M, Czedik-Eysenberg A, et al. (2009) A guide to using MapMan to visualize and compare Omics data in plants: a case study in the crop species, Maize. Plant, Cell & Environment 32: 1211-1229.
