# Supplementary material for: Ragweed (Ambrosia artemisiifolia) pollen allergenicity: SuperSAGE transcriptomic analysis upon elevated CO2 and drought stress
Source: BMC Plant Biol. 2014 Jun 27;14:176. doi: 10.1186/1471-2229-14-176 (PMC4084800; doi:10.1186/1471-2229-14-176)
Supplement: Additional file 7 — Correlation of SuperSAGE data with qRT-PCR data. 1–4: drought stress, 1: Amb a 1.1; 2: Amb a 1.2, 3: Amb a 1.3; 4: Amb a 9; 5–6: 700 ppm CO2 + drought, 5: Amb a 1.1; 6: Amb a 1.2. [file 1471-2229-14-176-S7.pdf]

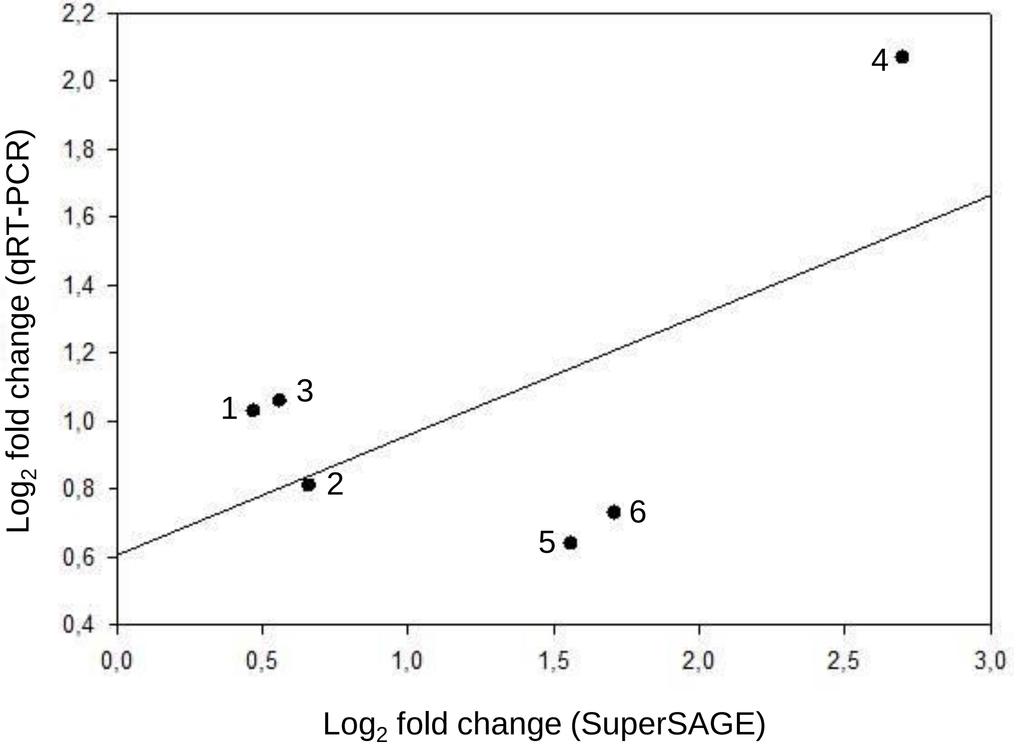

Additional file 7. Correlation of SuperSAGE data with qRT-PCR data. 1-4: drought stress, 1: Amb a 1.1; 2: Amb a 1.2, 3: Amb a 1.3; 4: Amb a 9; 5-6: 700 ppm CO_2 + drought, 5: Amb a 1.1; 6: Amb a 1.2. (R=0.59; constant variance test)
