# Supplementary material for: Ragweed (Ambrosia artemisiifolia) pollen allergenicity: SuperSAGE transcriptomic analysis upon elevated CO2 and drought stress
Source: BMC Plant Biol. 2014 Jun 27;14:176. doi: 10.1186/1471-2229-14-176 (PMC4084800; doi:10.1186/1471-2229-14-176)
Supplement: Additional file 8 — Greenhouse data. Temperature, relative humidity and light conditions in the greenhouse during the vegetation period of ragweed are given. [file 1471-2229-14-176-S8.pdf]

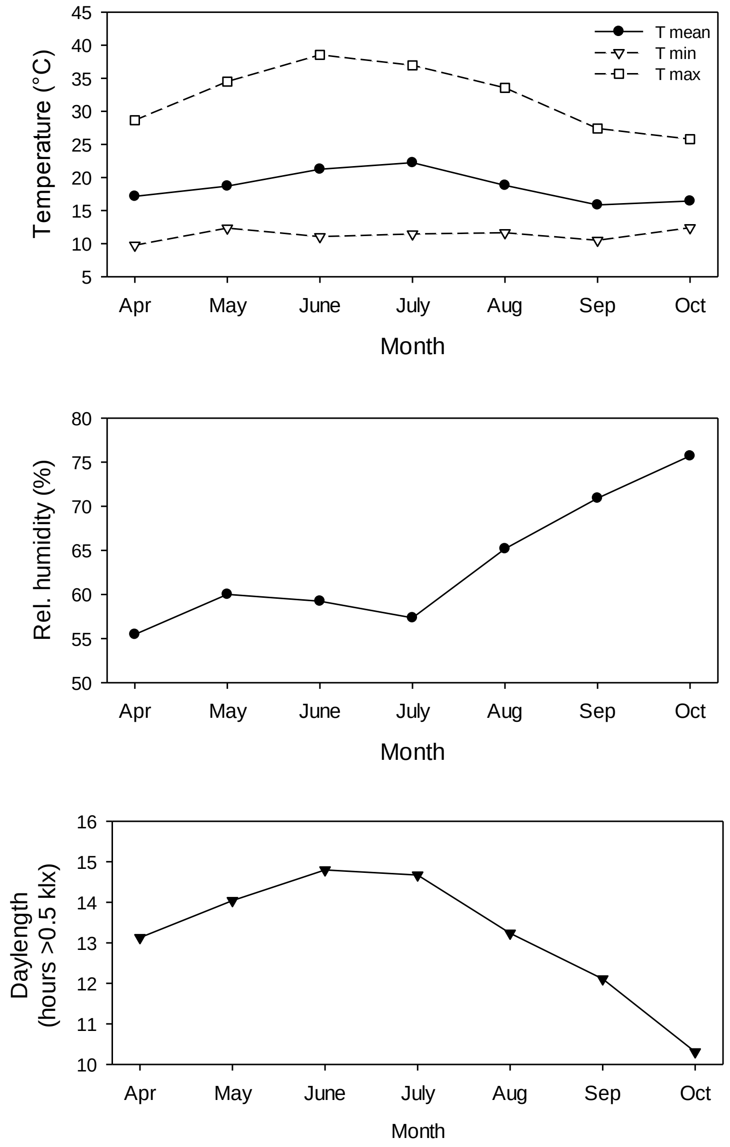

Additional file 8. Greenhouse data. Temperature, relative humidity and light conditions in the greenhouse during the vegetation period of ragweed are given.
