# Supplementary material for: Ragweed (Ambrosia artemisiifolia) pollen allergenicity: SuperSAGE transcriptomic analysis upon elevated CO2 and drought stress
Source: BMC Plant Biol. 2014 Jun 27;14:176. doi: 10.1186/1471-2229-14-176 (PMC4084800; doi:10.1186/1471-2229-14-176)
Supplement: Additional file 9 — Sequences of primers that were used for quantitative real-time RT-PCR (qRT-PCR). [file 1471-2229-14-176-S9.pdf]

Additional file 9. Sequence of primers used for quantitative real- time RT-PCR (qRT-PCR).

Gene	Acc. No.	Forward primer	Reverse primer
Amb a 1.1	M80558	5-GGGGCTGGTGACGAAAATATTG-3	5-CACCATGCCTTCCTAGGACATT-3
Amb a 1.2	M62981	5-TAACATCGTTAACGCCGGTCTCAC-3	5-TGATATCGAGCAGCCCATCGGAA-3
Amb a 1.3	M80560	5-GGTCGGGGAAATCTTACCTTCAGT-3	5-TGACCGTGTAGACATCACCCCATT-3
Amb a 1.4	M80562	5-TTTGACGAGCGAGGCATGCTAT-3	5-CTCTGACATGGCGGATTCACCATA-3
Amb a 1.5	M80561	5-GGAGCCAGAATGGATGACTTGGAA-3	5-TGTGGAACCATATCTCCCGGTTCA-3
Amb a 5	M84987	5-AGGATCCACAGATGAAGTCGATGA-3	5-AAACCACTTGCCAAGGACAGTACC-3
Amb a 6	U89793	5-GTTTCATGGAGGCCAACGATGTTC-3	5-GCCACACGATCAGCTTTGGTTT-3
Amb a 8	AY268427	5-AACCTGAGGAGATGAAAGGCA-3	5-GCTTGGCCTGTTTTCTTGATGC-3
Amb a 9	AY894657	5-AAGAATCTCGGCTCGGTGTCA-3	5-CTTGCCAACGTCCTTCATTAAGCC-3
α-tubulin	GW917730	5-TGCAGAGGGCTGTTTGCATGA-3	5-ACCCACGTACCAGTGAACAAAAG-3
